# The effect of smoking on clinical presentation and expression of TLR-2 and CD34 in Oral lichen Planus patients: clinical and immunohistochemical study

**DOI:** 10.1186/s12903-020-01118-2

**Published:** 2020-04-29

**Authors:** Nermine Raouf Amin, Nermin Yussif, Enji Ahmed

**Affiliations:** 1 Oral and Maxillofacial Pathology department, Faculty of Dentistry, Cairo University, Cairo, Egypt; 2 Oral Medicine and Periodontology Department, Faculty of Dentistry, Cairo University, Cairo, Egypt; 3 Oral Medicine and Periodontology Department, Faculty of Dentistry, Cairo University, Cairo, Egypt; 4 Faculty of Dentistry, British University in Egypt (BUE), El Sherouk City, Egypt

**Keywords:** Oral lichen planus, Smoking, Inflammation

## Abstract

**Background:**

Oral lichen planus is a chronic inflammatory disease which is considered as a potential precancerous condition. Numerous studies have confirmed that inflammation is a strong risk factor for cancer development. Smoking is associated with potentially malignant disorders of the oral and oropharyngeal mucosa. The adverse consequences of smoking in various pathologies are mediated by its effects on the immune-inflammatory system. Little is known about the influence of cigarette smoke content on the course of OLP and inflammatory response.

**Methods:**

Twenty oral lichen planus smoker patients, 20 oral lichen planus non-smoker patients and 20 control patients were included in this work. Pain and clinical scores were calculated for each patient. Image analysis to calculate area percent for TLR-2 and CD34 immuno-expression was performed. Data was tabulated and statistically analyzed.

**Results:**

The present study showed no statistically significant difference in clinical and pain scores between the smoker and non-smoker groups. However, there was a significant difference in area percent values for TLR-2 and CD34 immuno-expression between the smoker and the non-smoker groups.

**Conclusion:**

Smoking enhanced TLR-2 and CD34 expression in OLP which are considered as inflammatory mediators and are contributing factors in the pathogenesis of oral lichen planus.

## Background

Lichen planus is a common disorder in which auto-cytotoxic T-lymphocytes trigger apoptosis of epithelial cells leading to chronic inflammation [1]. It is considered to be a precancerous condition [2]. Chronic inflammation in OLP induces the expression of various cytokines which impacts cell migration, proliferation and differentiation, hence leading to cancer development [3]. Inflammation has been established by previous studies as a strong risk factor for cancer development [4, 5].

Toll-like receptors (TLRs) are members in the pattern-recognition receptors (PRRs) which recognize microbial antigens to which oral mucosa is continuously exposed. TLRs are triggered, not only by microbial structures, but also during tissue or cell damage [6] and enhance the inflammatory response [7].

Ohno et al. [8] found that TLR-2 was highly expressed in OLP tissues and may affect its pathogenesis. Moreover, Ng et al. [9] illustrated the role of TLR-2 in epithelial dysplasia.

Angiogenesis has an attentive role in the pathogenesis of chronic inflammatory diseases [10]. Alterations to angiogenesis using the endothelial cell marker CD34, have been implicated in the pathogenesis of OLP [11]. Moreover, CD 34 overexpression is considered a useful marker preceding oral cancer development as it increases from normal mucosa to dysplasia to carcinoma [12].

Smoking is associated with potentially malignant disorders of the oral mucosa [13]. According to the authors’ analysis, tobacco smoking increases the risk of OLP malignant transformation [14] as cigarette smoke contains substances that induce chronic inflammation at mucosal surfaces [15].

The purpose of this study was to evaluate the effect of smoking on clinical presentation and expression of TLR-2 and CD34 in OLP as markers of inflammation.

## Methods

### Patients’ selection

OLP patients were recruited from the out-patient clinic of Oral Medicine and Periodontology Department, Faculty of Dentistry, Cairo University. For control patients, tissue samples were taken from those undergoing operculectomy.

The aim of the present study is to compare the immunohistochemical expression of TLR-2 and CD34 in OLP between smokers and non-smokers. According to Klosek et al. [16] and Using G-power program, the effect size between both groups was found to be 2.16 using power of 80 and 5% significance level giving a total sample size of 15 patients (5 patients per group). This number was to be increased to a total sample size of 21 (7 patient per group) to compensate for possible losses during the follow up. The previous sample size was exceeded in this work to be 20 patients for each group.

All patients fulfilled the WHO’s clinical diagnostic criteria for OLP [17] which is the presence of bilateral, more or less symmetrical lesions and a lacelike network of slightly raised gray-white lines (reticular pattern). Erosive, atrophic, bullous and plaque-type lesions were accepted only as a subtype in the presence of reticular lesions elsewhere in the oral mucosa.

The patients were examined clinically using spot light and magnifying glass for oral lesions, and natural light for skin lesions.

All patients fulfilled the WHO’s histopathologic diagnostic criteria for OLP [18] which is the presence of a well-defined band-like zone of cellular infiltration (mainly lymphocytes) that is confined to the superficial part of the connective tissue, liquefaction degeneration in the basal cell layer and absence of epithelial dysplasia.

#### Inclusion criteria

Systemically free OLP patients as evaluated by the aid of the Dental Modification of the Cornell Medical Index to standardize their systemic condition [18] were included in this study. Both sexes, smokers and non-smokers were also included.

#### Exclusion criteria

Patients showing signs of malignancy as induration of the lesion, loss of flexibility or rolled edges and patients who had any other visible lesion than OLP were excluded from this study. Patients who had received any medication for at least 3 months before the biopsy taking except that for OLP, patients who had any systemic disease, pregnant and lactating women were also excluded from this study.

#### Ethical procedures

Each subject signed an informed written consent form. The Research Ethics Committee of Faculty of Dentistry, Cairo University revised and approved this research on 25/6/2019 with the number (19–6-30).

### Grouping of patients

Patients were divided into three groups; 20 individuals in each group as follows:

Group I: Control group.

Group II: Non-smoker patients with OLP.

Group III: Smoker patients with OLP.

III-Clinical assessment:

The following clinical criteria were evaluated for groups II and III.

#### Pain score

The symptomatology score was assessed using visual analogue scale (VAS), which consists of 10 scores in which the patient marked the point along the line that represented his pain. The scale was measured from no pain to the end of scale (0 = no pain, 10 = extremely painful) [19].

#### Clinical score

A clinical score was given to all OLP lesions during exacerbation according to the clinical severity on a scale that ranged from 0 to 5 according to the criteria set by Thongprasom et al., [20] as follows:

0: No lesion, normal mucosa.

1: White straie, no erythematous area.

2: White straie with atrophic area less than 1cm^2^.

3: White straie with atrophic area more than 1cm^2^.

4: White straie with erosive area less than 1 cm^2^.

5: White straie with erosive area more than 1 cm^2^.

Score for each patient was calculated by recording a score for each lesion in the oral cavity separately, then calculating the average of these scores.

### Biopsy taking

Biopsy was taken from OLP lesions. For small lesions (< 4–5 mm in diameter), an excisional biopsy was taken. For large lesions (> 4-5 mm in diameter), an incisional biopsy was performed taking part of the lesion with part of the adjacent normal mucosa [21]. Tissue samples were obtained under ring block anesthesia from all groups and were placed immediately in 10% neutral buffered formalin fixative.

### Histopathological preparation

A Hematoxylin and Eosin (H&E) stained slide was prepared to confirm the diagnosis. Two sections were mounted on positively charged slides. One for the application of the primary antibody and the other one served as a negative control.

### Immunohistochemical staining

#### Antibodies (TLR-2 and CD34)

TLR-2 antibody (mouse monoclonal primary antibody, sc-21,759, Santa Cruz Biotechnology, Santa Cruz, CA, USA) diluted at 1:100 and a ready to use CD 34 (mouse monoclonal primary antibody, AM353-5 M, Biogenex, USA) were used in this work.

### Steps of basic immunostaining procedures [22]

Immunostaining for TLR-2 and CD34 was performed using Ventana Bench mark autostainer (Ventana Medical Systems, Tucson, AZ, USA) at Pathology Department, National Cancer Institute; Cairo University, as follows:

Deparaffinization and hydration of the tissue sections were done in descending grades of alcohol each for 10 min. Tissue sections were boiled in 10 mM citrate buffer, pH 6.0 for 10–20 min followed by cooling at room temperature for 20 min (antigen retrieval step). The sections were then incubated in 0.3% hydrogen peroxide for 30 min to block the endogenous peroxidase activity. The sections were washed before the application of 100 ml of TLR-2 antibody at dilution of (1:100) under incubation temperature of 30 °C for 80 min and CD34 antibody under incubation temperature of 30 °C for 20 min, followed by application of the secondary antibody for 30 min. Diaminobenzidine tetrahydrochloride (DAB) was applied to sections for 15 min at room temperature. Sections were counterstained with Mayer’s Hematoxylin which was applied for 8 min and then a bluing reagent was applied for 4 min. Slides were extracted and arranged in racks. They were washed in tap water for 5 min and then dehydrated in ascending grades of alcohol each for 5 min. Slides were cleared in xylene and then cover slips were applied and mounted using Distyrene Plasticizer Xylene (DPX) mounting agent.

N.B. Immunohistochemical staining was carried out in one batch for standardization.

### Immunohistochemical assessment

#### Transmission light microscope

The immunostained sections were examined using low and high power fields by the light microscope.

#### Image analysis computer system

The image analyzer computer system applying the software Leica Quin 500* {Leica Microsystems LTD. CH9435 Meerbrugg Type: DFC295 (12730469), Input:12v/170 MA, Serial number: 0557060916, Switzerland} was used for measuring the area percent of positive TLR-2 and CD34 immunoexpression in high power fields (× 400 magnification). Area percent was calculated from three fields per patient. Fields were randomly chosen from well stained sections that properly represented the histopathology of OLP. Mean area percent values for both markers were calculated for the studied groups.

### Statistical analysis

The obtained data was presented as mean ± standard deviation (SD) values. *P* values< 0.05 were considered significant. Statistical analysis was performed by using a computer program IBM SPSS. Student t-test was used to compare between two groups regarding the clinical and pain scores. One Way Analysis of Variance (ANOVA) test was used to compare between three groups followed by Tukey’s post hock test for pairwise comparison between each two groups regarding TLR-2 and CD34 immunoexpression.

## Results

### Clinical presentation

The mean clinical and pain score values for the smoker LP group were (4.66 ± 0.02 &6.32 ± 0.13, respectively), while for the non-smoker LP group, they were (4.64 ± 0.04& 6.26 ± 0.55, respectively) showing no statistically significant difference between the two groups (*p* = 0.06&0.12, respectively).

### Microscopic examination of immunostained sections

#### TLR-2

**Fig. 1:**
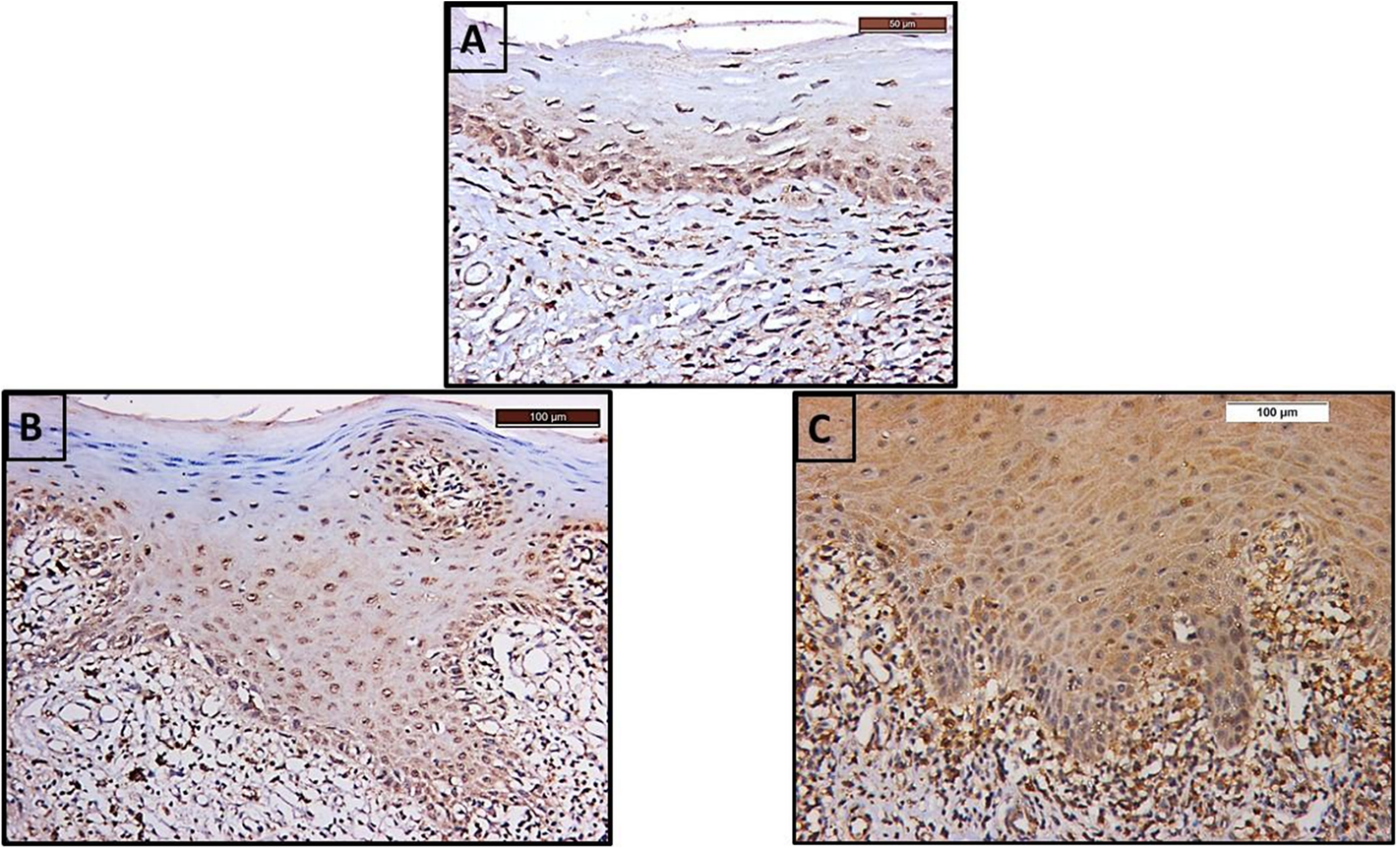
Photomicrographs of TLR-2 immunoexpression in the epithelium of the **a** control group showing cytoplasmic reaction in the basal and suprabasal cells (TLR-2 × 400), **b** non-smoker LP group showing cytoplasmic and nuclear reaction in the basal and prickle cells (TLR-2 × 200), **c** smoker LP group showing obvious diffuse cytoplasmic reaction in all layers of stratified squamous epithelium (TLR-2 × 200). (LP): Lichen planus

Cytoplasmic TLR-2 immunoexpression was seen in basal and suprabasal cells of stratified squamous epithelium of the control group. (Fig. 1a).

Buccal mucosa of non-smoker LP group showed cytoplasmic TLR-2 immunoexpression in the basal and prickle cells, but not in the granular and surface layers. Nuclear expression is also detected. (Fig. 1b). Buccal mucosa of smoker LP group revealed obvious diffuse cytoplasmic TLR-2 immunoexpression in all layers of stratified squamous epithelium. (Fig. 1c).

#### Cd 34

**Fig. 2:**
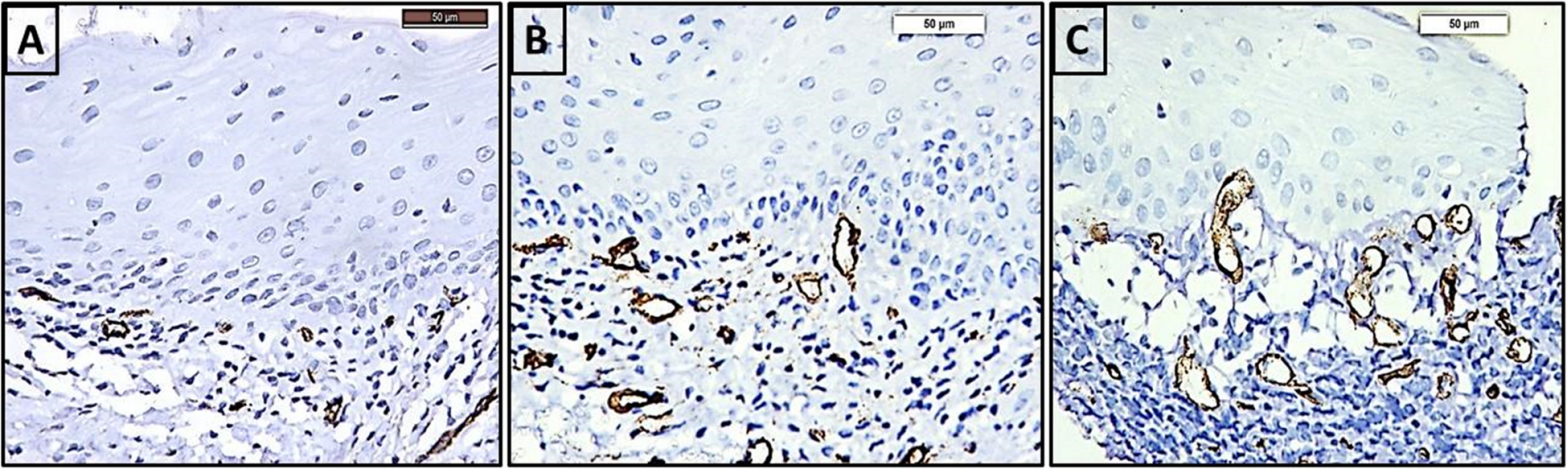
Photomicrographs of CD34immunostained sections in the mucosa of the **a** control group showing few blood vessels, **b** non-smoker LP group showing numerous and elongated blood vessels **c** smoker LP group showing numerous and rounded blood vessels. (CD34 × 400). (LP): Lichen planus

Cytoplasmic CD 34 immunoexpression was seen in endothelial cells lining blood vessels in the connective tissue of all groups. In the control group (Fig. 2a), blood vessels were few and small in size. In the non-smoker LP group (Fig. 2b), more blood vessels were seen, appeared more elongated and were irregularly distributed. In smoker LP group (Fig. 2c), blood vessels were numerous, most of them were rounded and were irregularly distributed.

### Statistical analysis

ANOVA test revealed that the difference between the three groups for TLR-2 and CD34 immunoexpression was statistically significant (*P* < 0.05) (Table 1). Tukey post hock test for pairwise comparison between each two groups revealed that the mean area percent of TLR-2 and CD34 immunoexpression in the smoker LP group was significantly greater than the non-smoker LP group (*P* < 0.01) and the control group (*P* < 0.01). The non-smoker LP group was significantly greater than the control group (*P* < 0.01). (Table 1).

**Table 1:** Mean area percentage of TLR-2 and CD 34 immuoexpression in the control, non-smoker LP and smoker LP groups. (ANOVA and Tukey Post hock tests)

Groups	Control group	Non-smoker LP group	Smoker LP group	***P*** value
**Point of comparison**				
**TLR-2** **Mean ± SD** ***n*** **= 20**	14.03 ± 0.59^a^	37.76 ± 1.41^b^	67.67 ± 1.51^c^	*P* < 0.05
**CD34** **Mean ± SD** ***n*** **= 20**	2.37 ± 0.04^a^	6.05 ± 0.03^b^	8.50 ± 0.06^c^	*P* < 0.05

*P* value < 0.05 is considered significant

Values having different letters are significantly different

## Discussion

In this work, clinical examination revealed that both groups whether smokers or non-smokers showed the classical presentation of OLP and this was confirmed by calculating the mean clinical score values which showed no significant difference between both groups. This is in accordance to Gorsky et al. [23] who showed no difference in the clinical type or symptoms of OLP between smokers and non-smokers. This can be explained by the fact that many of the patients in both groups had reticular type with low clinical score values. Moreover, Gorsky et al. [23] found no statistical association for the atrophic form of OLP with the presence and intensity of symptoms.

This study revealed an insignificant difference in pain score values between smokers and non-smokers. Some of the cases were reticular and smoking may not cause sensitivity of the oral mucosa in reticular OLP. Moreover, smoker patients with atrophic or erosive types tend to decrease the frequency of smoking to reduce irritation caused by heat and out of fear of possible malignant transformation based on previous knowledge about the relation between smoking and oral cancer.

In our results, microscopic examination of TLR-2 immunostained sections revealed positive TLR-2 reaction in normal epithelial cells of the control group. Hill and Diehl [24] declared that, in humans, TLR expression is mainly expressed in immune cells, where it drives immune responses and is less widespread in epithelial cells where it offers a barrier against pathogens.

TLR-2 was expressed in basal cells of normal epithelium. This is in accordance to Ohno et al. [8] who revealed high expression in basal keratinocytes of the normal buccal mucosa. This finding could be explained by Salem et al. [25] who pointed out that the outmost epithelial layers depend on their junctional attachments for defense not needing to express TLRs whereas the deeper basal cells use their TLRs to provide immunologic backup.

OLP patients in this study, whether smokers or non-smokers, expressed TLR − 2 in basal as well as in spinous cell layers. This is in accordance to Ohno et al. [8] who revealed high expression in basal and spinous layers in OLP patients. Salem et al. [25] revealed that the integrity of oral epithelium is disrupted in OLP thus paves the way for pathogen activated molecular patterns (PAMPs) to diffuse into deeper epithelial layers causing irritation to more superficial epithelial layers, so TLR-2 expression in OLP extended from basal to spinous layers to combat the invading allergens. This could also be attributed to the nature of TLRs which are members of PRRs that are triggered, not only by microbial structures, but also during tissue or cell damage as revealed by Takeuchi and Akira [6]. The damaged epithelial cells in OLP recruit inflammatory cells which release cytokines [3] and activate TLRs expression and enhance the inflammatory response [7].

OLP cases, whether smokers or non-smokers, showed cytoplasmic TLR-2 immunoexpression. Uronen-Hansson et al. [26] declared that TLR-2 is highly expressed in the cytoplasm in a perinuclear region very close to Golgi complex associated with microtubules which serve as transport tracks for TLR-2 vesicles. Statistical analysis of the present results revealed that the mean area percent of TLR-2 immunoexpression in the epithelium of OLP patients whether smokers or not was significantly greater than the control group. This was previously illustrated by Ohno et al. [8] who declared that the number of TLR-2 transcripts was increased in OLP compared to normal gingival tissues as indicated by real time- polymerase chain reaction (RT-PCR) and verified their work immunohstochemically.

The previous finding is supported by Liu et al. [27] who revealed that TLR-2 expression was augmented in OLP by cytokines. These results suggest that TLR-2 may be involved in the pathogenesis of OLP.

Moreover, smoking OLP patients showed significantly greater mean area percent values for TLR-2 immunoexpression in the epithelium compared with non-smoker OLP patients. Johnson et al. [28] supported our findings and explained that tissues exposed to tobacco carcinogens responded by expressing elevated levels of cytokines as part of response to injury. Therefore, we could speculate that smoking resulted in enhanced cytokine release which led to activated TLR-2 inflammatory signaling.

Cytoplasmic CD34 immunoexpression was seen in endothelial cells lining blood vessels in the connective tissue of all groups. In the control group, blood vessels were few and small in size. In the non-smoker OLP group, more blood vessels were seen, appeared elongated and were irregularly distributed. In smoker OLP group, blood vessels were numerous, most of them were rounded and were irregularly distributed. This is in accordance with Klosek et al. [16] who observed few blood vessels in the control group and numerous elongated irregularly distributed blood vessels in the non-smoker OLP group. They also revealed that smoking in OLP increased the number of blood vessels which were small in size.

Mean area percent of CD34 immunoexpression in OLP patients whether smokers or non-smokers was greater than the control group. This was in accordance to Tao et al. [10] whose results documented an increase in the mean vascular density in OLP group compared to control group. Mittal et al. [11] found that the mean vascular density in OLP group stained by CD34 was significantly greater than the control group showing increased angiogenesis in the erosive OLP form compared to the reticular form. The previous results indicated that angiogenesis was closely correlated to OLP lesions.

Smoking in our study enhanced angiogenesis in OLP as confirmed by enhanced CD34 immunoexpression in OLP patients. Klosek et al. [16] previously noted a significant increase in blood vessel density stained by CD34 in smoking OLP patients compared to non-smoker patients. They related their results to the effect of smoking on enhancing the release of pro-inflammatory cytokines.

## Conclusion

Collectively our results suggest that smoking enhanced TLR-2 and CD34 expression in OLP which are considered as inflammatory mediators and are contributing factors in the pathogenesis of OLP.

## Data Availability

The data that support the findings of this study are available from faulty of Dentistry-Cairo University but restrictions apply to the availability of these data, which were used under license for the current study, and so are not publicly available. Data are however available from the authors upon reasonable request and with permission of faculty of Dentistry-Cairo University.

## Abbreviations

OLP: Oral lichen planus
TLR: Toll-like receptor
CD: cluster of differentiation
PRRs: Pattern-recognition receptors
WHO: World health organization
VAS: Visual analogue scale
H&E: Hematoxylin & Eosin
DAB: Diaminobenzidine tetrahydrochloride
DPX: Distyrene plasticizer xylene
ANOVA: One way analysis of variance
